# Retrospective Analysis of Dermal Absorption Triple Pack Data

**DOI:** 10.14573/altex.2101121

**Published:** 2021-03-12

**Authors:** David G. Allen, John Rooney, Nicole Kleinstreuer, Anna Lowit, Monique Perron

**Affiliations:** 1 Integrated Laboratory Systems LLC, Research Triangle Park, NC, USA; 2 National Toxicology Program Interagency Center for the Evaluation of Alternative Toxicological Methods, National Institute of Environmental Health Sciences, Research Triangle Park, NC, USA; 3 Office of Pesticide Programs, U.S. Environmental Protection Agency, Washington, DC, USA

## Abstract

Dermal toxicity is driven by the ability of a substance to penetrate the skin. The “triple pack” approach, which combines *in vivo* rat, *in vitro* rat, and *in vitro* human data, is used to calculate an estimated human dermal absorption factor (DAF). To assess the feasibility of deriving a DAF using only *in vitro* data, we retrospectively evaluated agrochemical formulations to compare the DAF derived from each individual method to the DAF generated from the triple pack approach. For most of the formulations evaluated, the *in vitro* rat method generated a similar or higher DAF value than the *in vivo* method. Absorption through *in vitro* human skin was similar to or less than that observed in rat skin for all formulations. For most of the formulations, the human *in vitro* method provided a similar or higher estimate of dermal absorption than the triple pack approach. For human health risk assessment, *in vitro* assays using human skin would be preferable, as they would be directly relevant to the species of interest and avoid overestimation of dermal absorption using rat models. However, rat *in vitro* studies would still have utility in the absence of human *in vitro* data. *In vitro* rat data provide estimates of dermal absorption that are at least as protective as *in vivo* rat data and thus could also be considered adequate for use in establishing DAFs. The comparisons presented support potentially using *in vitro* data alone for DAF derivation for human health risk assessment of pesticides.

## 1 Introduction

To support registration and sale of agrochemical products, regulatory authorities require characterization of potential risks associated with exposure to these pesticides through all possible routes, including the dermal route. Dermal toxicity is primarily driven by the extent to which an agrochemical penetrates the skin and is subsequently absorbed into the systemic circulation. In some circumstances, data from oral toxicity studies are used to evaluate dermal exposures. In such cases, a dermal absorption factor (DAF) is used to convert oral doses to dermal-equivalent doses (i.e., route-to-route extrapolation).

Historically, regulatory authorities have relied on results from guideline *in vivo* dermal absorption studies in rats to estimate a DAF (EPA, 1998; OECD, 2004a). Rat skin is more permeable than human skin due to anatomical differences such as thickness and concentration of hair follicles and thus provides a conservative estimate of skin absorption that might occur in humans. *In vitro* methods using both rat and human skin are also used to measure dermal absorption and likewise are conducted in accordance with an internationally harmonized test guideline (OECD, 2004b). With the advent of *in vitro* methods using both rat and human skin, a refined estimate of human dermal absorption was developed. The so-called “triple pack” combines results from *in vivo* rat, *in vitro* rat, and *in vitro* human studies to calculate an estimated human DAF as described by the following equation:

triple pack DAF=rat in vivo×(human in vitro÷rat in vitro)

The original underlying assumption of the triple pack was that a ratio of 1 would be obtained when comparing *in vitro* and *in vivo* rat absorption. This suggested that when the same technique is used with human skin *in vitro*, such results would likely be a good predictor of human *in vivo* dermal absorption. However, as more experience has been gained with *in vitro* studies, this expectation of equivalent results from *in vivo* and *in vitro* rat studies has become recognized as unattainable in most cases since the studies are inherently different. Operational differences between *in vivo* and *in vitro* studies that can lead to differing results include binding of the test substance to the protective covering *in vivo*; a semi-occlusive environment *in vivo*; and skin being hyperhydrated from the receptor fluid in *in vitro* assays. Regardless, the triple pack approach still provides a quantitative way to address both species differences and differences between the *in vivo* and *in vitro* assays.

With ever-increasing emphasis on transitioning away from laboratory animal studies to more human-relevant approaches to predicting potential toxicity, there is widespread interest in moving to a fully *in vitro* approach to evaluating dermal absorption of agrochemicals. However, regulatory authorities need to have sufficient confidence that such an approach will not greatly underestimate dermal absorption in humans. Concern over the reliability and consistency of the *in vitro* results prompted convening of a 2012 workshop to examine and define best practices for conducting *in vitro* dermal absorption studies (Sullivan et al., 2017). Workshop participants recommended specific study reporting practices to increase consistency of data included in regulatory submissions. More recently, an international panel of experts convened in September 2019 in Dublin to discuss the available skin absorption test methods and their associated Organisation of Economic Cooperation and Development (OECD) test guidelines and available guidance documents (Hopf et al., 2020). Discussions emphasized the continued challenges associated with such key methodological elements as diffusion cell systems, receptor fluid, sampling, skin thickness, test substance application, terminology, and data interpretation.

Guidance documents from OECD and the European Food Safety Authority (EFSA) (OECD, 2011; EFSA, 2017) are now available that advocate for the utility of *in vitro* human skin in human health risk assessments. These serve as the basis for the acceptance of *in vitro* data for registration by European authorities. However, since 2008, regulatory authorities of countries participating in the North American Free Trade Agreement (NAFTA) have considered the triple pack as the preferred approach to refine estimates of dermal absorption by incorporating *in vitro* data. Such an approach was also intended to standardize and improve the quality of *in vitro* studies. Conducting such studies would also generate a comparative database that could allow a retrospective evaluation of all three study types (i.e., *in vivo* and *in vitro* rat, *in vitro* human) to evaluate the potential of relying upon *in vitro* studies for deriving DAFs for human health risk assessment (NAFTA, 2008; Sullivan et al., 2017).

The experts at the 2012 workshop encouraged collaboration among regulatory authorities and industry stakeholders to facilitate the use of *in vitro* dermal absorption methods for risk assessment. They recommended that the dataset envisioned by the NAFTA working group be compiled and analyzed. To that end, the National Toxicology Program Interagency Center for the Evaluation of Alternative Toxicological Methods (NICEATM) and the U.S. Environmental Protection Agency (EPA) Office of Pesticide Programs (OPP) have worked with agrochemical companies to assemble a comprehensive dataset of available triple pack study results.

This paper describes the results of a retrospective evaluation of triple pack data that focused on assessing the difference in dermal penetration between rat and human skin, and how estimates of skin permeability generated using the triple pack differed from estimates generated using *in vitro* human skin data alone.

## 2 Materials and methods

### 2.1 Dermal absorption test methods

As noted above, there are harmonized test guidelines with detailed protocol information for each of the methods included in this data analysis of existing studies. A brief overview of each method is provided below.

#### In vivo *rat method*

For the *in vivo* rat method (EPA, 1998; OECD, 2004a), dermal absorption is measured following topical exposure of a finite dose of test substance to intact skin. The guidelines recommend use of at least three dose levels that span the range of expected field exposures. Suggested exposure durations are 10 h in the EPA guideline and 6 or 24 h in the OECD guideline. Skin samples are typically collected immediately following a post-application washing and then at post-application time points (e.g., 0.5, 1, 2, 4, 10 and 24 h, and then daily up to 7 d after the final dose interval). Accordingly, for the 30 studies included in our analysis, exposure times ranged from 6 to 10 h. While the duration of the studies ranged from 1 to 21 days, most of the studies (26/30) ranged from 5 to 7 days. A total material balance was obtained from each animal, and total compound was obtained from each of the following: wash and any obtained on materials (e.g., o-ring, gauze), urine, feces, cage wash, blood, skin application site, residue in the carcass, and stratum corneum as represented by up to 20 sequential tape strips (although this assessment was not performed in two of the studies).

#### In vitro *methods*

For the *in vitro* rat and human methods (OECD, 2004b), dermatomed skin (approximately 200–400 µM thick) is typically used. Skin samples are placed on a diffusion cell and treated with at least two finite dose levels that span the realistic range of human exposures. Either static or flow-through systems are considered acceptable with the following stipulations: For flow-through systems, the rate of flow must not hinder diffusion. For static systems, the receptor fluid must be continuously stirred and sampled regularly. While the guideline provides for sample collection at intervals up to 24 h, our analysis focused specifically on the final sample collected at the 24 h study termination point. As in the *in vivo* system, a total material balance is obtained from each sample, which includes assessing the amounts of test material in the receptor solution, wash, skin, and final cell wash.

### 2.2 Data compilation

Data used in this analysis were compiled from a variety of sources. EPA provided NICEATM with data evaluation records and other documents used during OPP registration and risk assessment reviews. To ensure that the data used in the analyses were generated from standardized test method protocols and best reflected the recommendations on harmonized reporting requirements, only studies conducted since 2010 were considered. Triple pack study reports were also provided to NICEATM by ADAMA, BASF, Bayer, Corteva, FMC, Syngenta, and Valent. A total of 30 agrochemicals had data from *in vivo* rat, *in vitro* rat, and *in vitro* human test methods that met the criteria for inclusion in the dataset. Table 1 provides the breakdown of formulation types represented by these 30 agrochemicals. We also collected physicochemical property information to use in comparisons across the 30 substances. Because the complete formulation components are business confidential and thus not available for analysis, physicochemical property information was limited to those properties associated with the complete formulations (i.e., the formulation type as listed in Tab. 1) and their associated active ingredients. For the active ingredients, physicochemical properties relevant to dermal absorption (i.e., molecular weight, water solubility, vapor pressure, octanol-water partition coefficient) were compared across chemicals for which such information was available. Property values were obtained using the Open Structure-Activity/Property Relationship App (OPERA) as provided in the Integrated Chemical Environment v3.2^1^.

All extracted data were reviewed by two individuals and any discrepancies reconciled prior to conducting analyses. Table 2 lists the data types that were collected from each data evaluation record or study report.

### 2.3 Dermal absorption measurements

For *in vivo* studies, dermal absorption was calculated as either direct absorption or potential absorption, both of which were expressed as a percentage of the applied dose. Consistent with the OECD test guideline (OECD, 2004a), direct absorption was represented by the amount of test substance recovered in urine, feces, cage wash, blood, carcass, and the skin exclusive of application site. Potential absorption was expressed as the sum of direct absorption and the amount of chemical measured at the skin application site and in the stratum corneum. The amount of test substance in the stratum corneum was estimated using tape strips. A conservative approach includes all tape strips, but the more common approach excludes the first two tape strips, assuming that this surface material would not be absorbed and measures test substance on all subsequent tape strips. *In vitro* dermal absorption was represented by the amount recovered in the receptor fluid and the amount of substance retained in the skin (i.e., dosed skin sample, including the stratum corneum with tape strips treated the same as above, if performed).

### 2.4 Dermal absorption comparisons

**Fig. 1:**
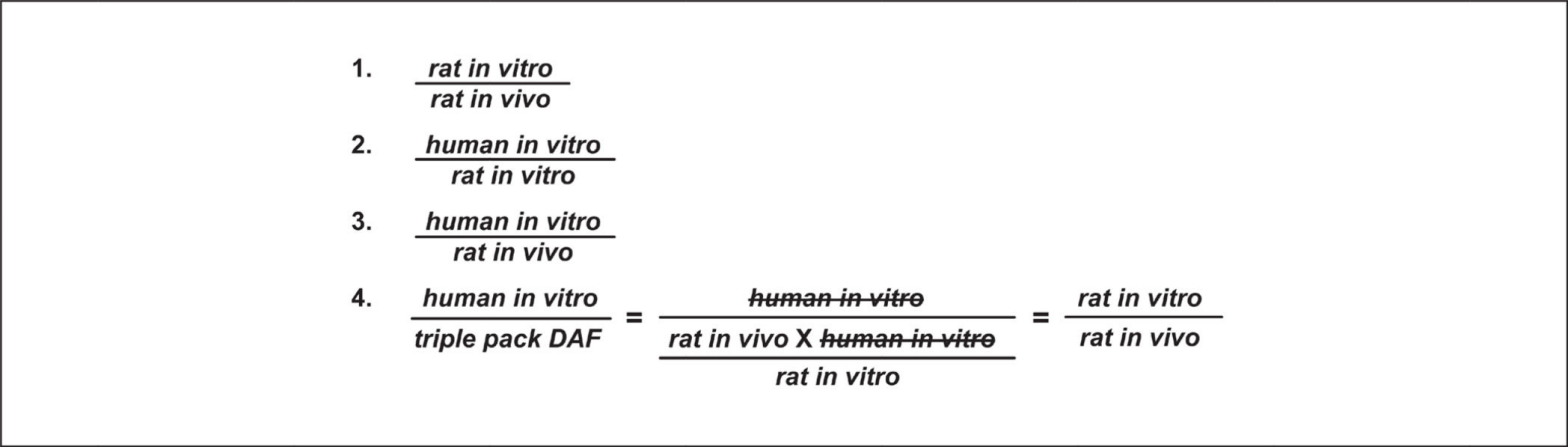
Absorbance ratios calculated for comparisons

The mean and standard deviation (SD) for each absorption value, derived as described in the previous section for *in vitro* rat, *in vitro* human, and *in vivo* rat studies, were calculated to enable comparison among dermal absorption test methods. Dermal absorption values measured in *in vitro* human and rat test methods were compared to one another, and each of these was also compared to *in vivo* rat dermal absorption (Fig. 1, Equations 1, 2, and 3). *In vitro* rat and human comparisons were matched by dose and time. Comparisons of data from both *in vitro* methods to *in vivo* rat data were also time-matched (24 h). Additional comparisons were based on the longest *in vivo* time point (up to 7 days) and used either potential or direct *in vivo* dermal absorption values. As noted above, two separate potential absorption values were calculated based either on inclusion of all tape strips or all tape strips except the first two. Finally, the DAF calculated based on results from the triple pack was compared to the DAF generated using only *in vitro* human data. This was done by calculating the ratio between the *in vitro* human DAF and the triple pack DAF, which is mathematically equivalent to the ratio of rat *in vitro* to rat *in vivo* results (Fig. 1, Equation 4). Results were organized into tables to provide a direct comparison of a DAF established solely based on *in vitro* human data to the DAF derived from the triple pack.

### 2.5 Accounting for test method variability in dermal absorption comparisons

**Fig. 2:**
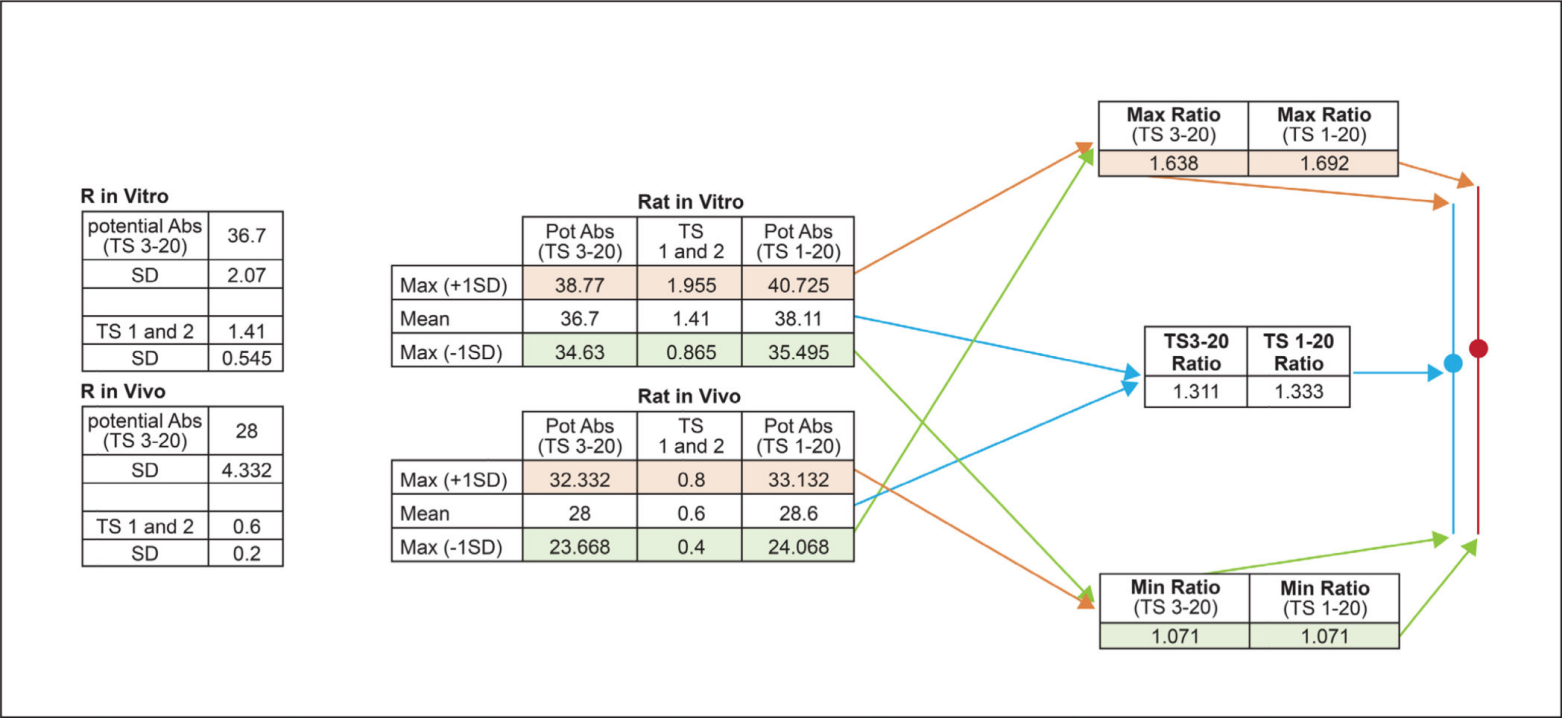
Calculating the impact of variability on absorbance ratios Maximum and minimum ratios were calculated to establish the range of possible outcomes. The maximum ratio is the sum of the mean and SD of replicate measurements in the numerator and the difference of the mean and SD in the denominator. The minimum ratio is based on the difference of the mean and SD in the numerator and the sum of the mean and SD of replicate measurements in the denominator. TS, tape strips; Pot Abs, potential absorption (the sum of direct absorption and the amount of chemical measured at the skin application site and in the stratum corneum).

Assay variability is an important consideration in evaluating and comparing the performance of different testing approaches. To evaluate the impact of variability in each of the test methods on the comparisons, three separate ratios were calculated for each of the comparisons in Figure 1. Mean absorbance values were used to calculate an initial ratio. A “maximum ratio” was calculated as the ratio of the sum of the mean and SD of replicate measurements in the numerator and the difference of the mean and SD in the denominator. A “minimum ratio” was calculated as the inverse of this ratio, i.e., with the difference of the mean and SD in the numerator and the sum of the mean and SD of replicate measurements in the denominator. Figure 2 illustrates these calculations with a comparison of the *in vitro* rat to *in vivo* rat methods.

## 3. Results

### 3.1 Comparison of dermal absorption in *in vitro* and *in vivo* rat test methods

In general, *in vitro* rat absorption was approximately equal to or greater than *in vivo* rat absorption, as indicated by a ratio greater than or equal to 1. This would make estimates of absorption derived from the *in vitro* assay more conservative than those derived from the *in vivo* assay.

**Fig. 3:**
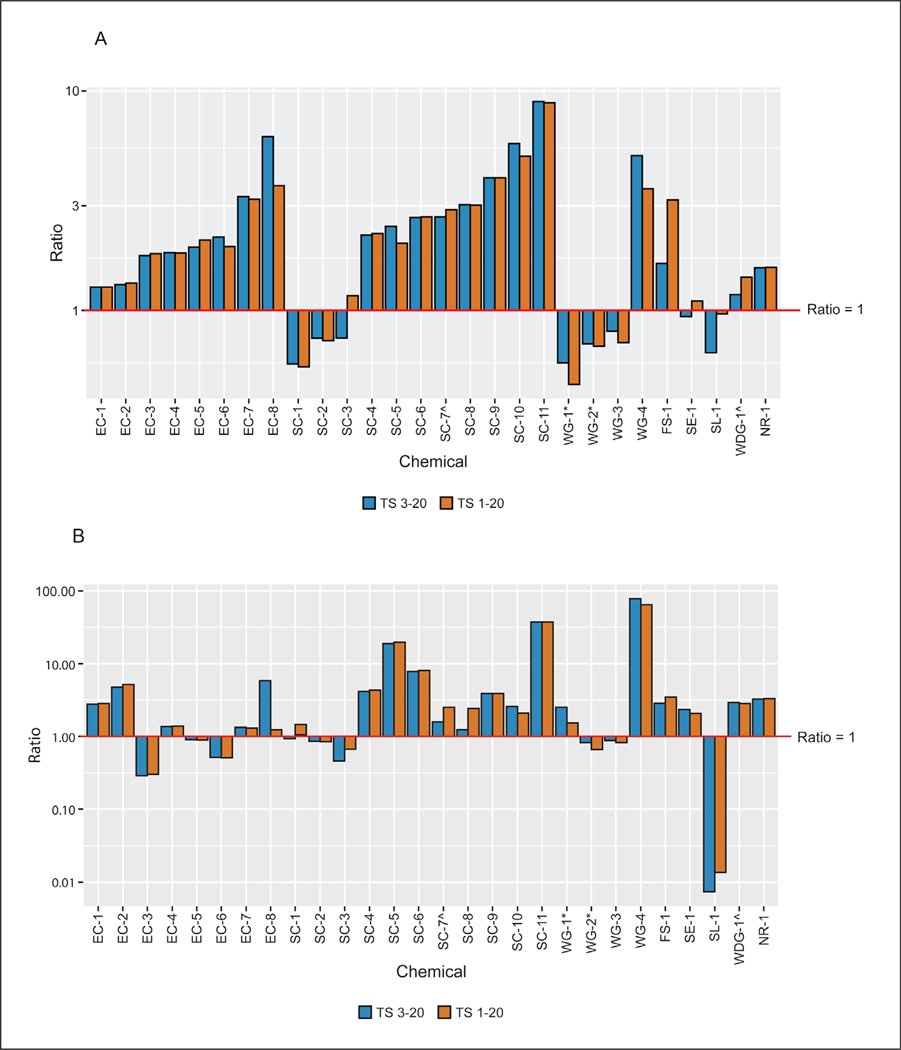
Dermal absorption time-matched comparisons at 24 h: *in vitro* rat vs *in vivo* rat (A) Low dose group; (B) high dose group. TS, tape strips. ^ and * indicate formulations that include the same active ingredient in a different formulation.

**Fig. 4:**
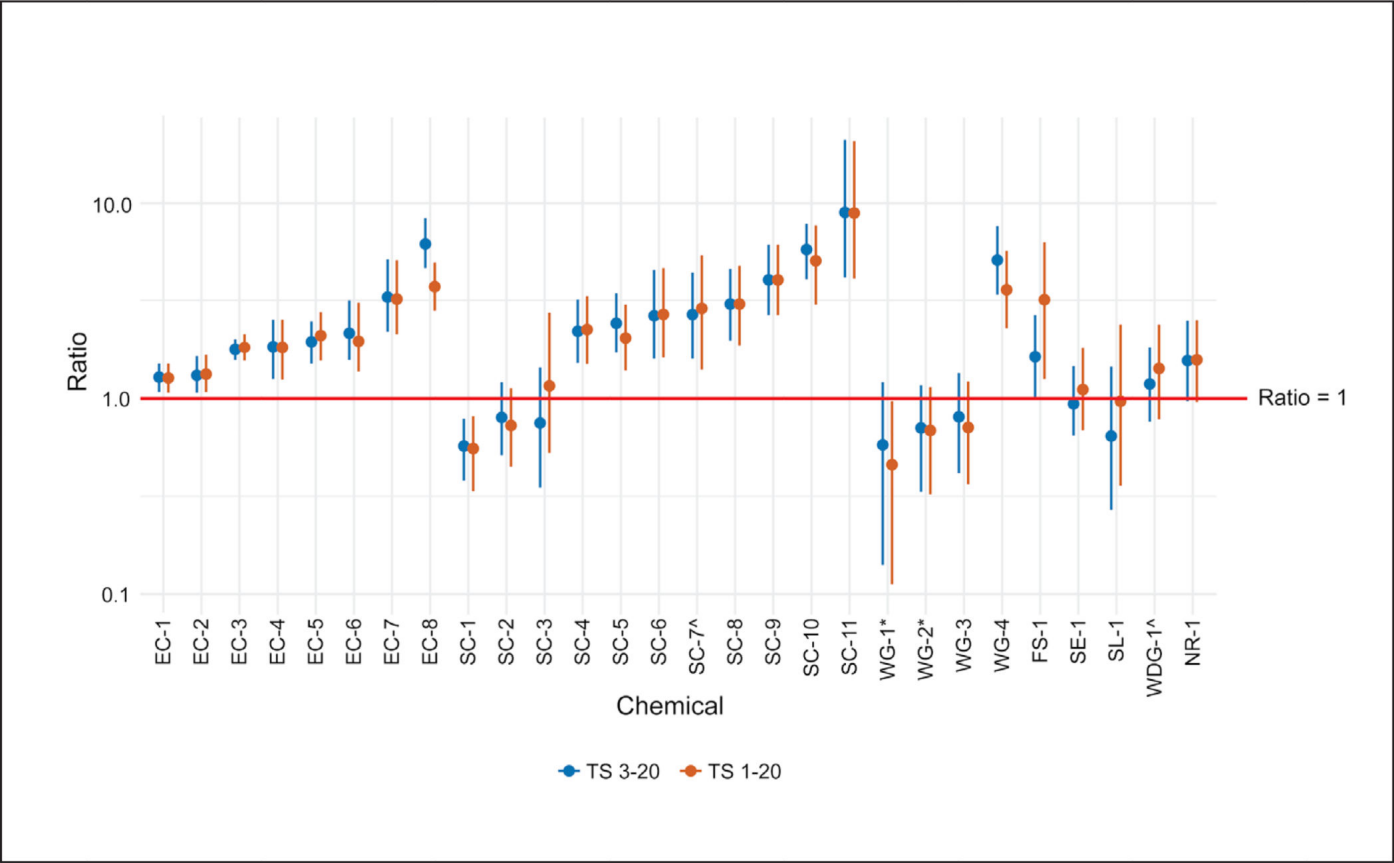
Impact of variability on absorbance ratios Range of possible absorbance ratios based on the maximum and minimum ratios calculated with or without tape strips (TS) 1 and 2. Data presented are from low dose ratios of *in vitro* and *in vivo* rat data, time-matched at 24 h. Dots are mean absorbance ratios and each end of the line is the maximum (top) and minimum (bottom) absorbance ratio. See Figure 2 for an example of how these data were calculated. ^ and * indicate formulations that include the same active ingredient in a different formulation.

Time-matched comparisons of *in vitro* and *in vivo* rat test method data at the 24 h time point were made for 28 of the formulations for which such data were available. For 71% (20/28) of these formulations, the ratio of *in vitro* to *in vivo* potential absorption for the low dose groups (calculated using tape strips 3–20) indicated that the rat *in vitro* result was more conservative than the rat *in vivo* result (Fig. 3A). Ratios for these 20 substances ranged from 1.18 to 8.94 (only 6/20 had ratios greater than 3), while the ratios for the remaining eight substances ranged from 0.57 to 0.94. When all tape strips were included in the calculation of potential absorption, which represents a more conservative approach, only six substances had an *in vitro* absorption value less than the *in vivo* absorption value, with calculated *in vitro* to *in vivo* ratios ranging from 0.46 to 0.96. The remaining 22 formulations had ratios ranging from 1.10 to 8.83 (with 7/22 having ratios greater than 3) (Fig. 3A). In either scenario, when variability of responses in both assays was considered, 96% (27/28) of the substances would have had an *in vitro* to *in vivo* absorption ratio greater than 1 regardless of the number of tape strips included (Fig. 4).

Similar results were noted for the high dose group (Fig. 3B). At high doses, three formulations had extremely high absorption through *in vitro* skin, resulting in very large (greater than 18-fold) absorption ratios when compared to *in vivo* rat skin. We evaluated these three substances and associated studies in more detail to determine whether physicochemical properties and/or any test method protocol components (e.g., vehicle, formulation type, etc.) could help explain these larger differences, but we found no such associations.

**Fig. 5:**
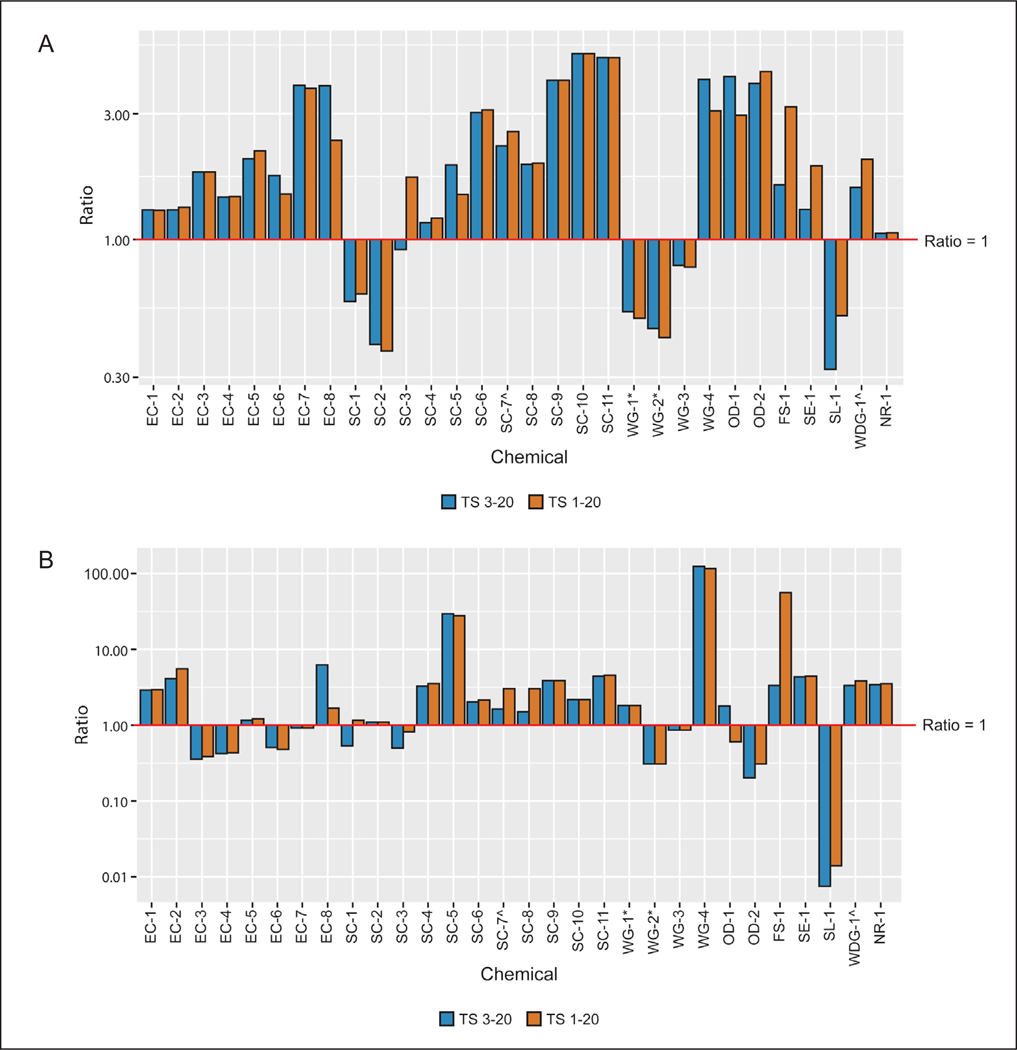
Dermal absorption comparisons: *in vitro* rat vs *in vivo* rat, maximum *in vivo* time point (A) Low dose group; (B) high dose group; ^ and * indicate formulations that include the same active ingredient in a different formulation. TS, tape strips.

Ratios calculated using the longest *in vivo* timepoint (which ranged from 24 hours to 7 days post-dosing) also indicated that absorption estimates derived from rat *in vitro* data would be more conservative than those derived from rat *in vivo* data. For the low dose group, 77% (23/30) of the formulations evaluated had ratios ranging from 1.05 to 5.07 (Fig. 5A). The seven formulations that had a ratio of less than 1 (ranging from 0.32 to 0.92) also had a ratio less than 1 in the time-matched comparisons.

We identified no common property among the substances having ratios less than 1 that would explain why this result was observed. However, it should be noted that 75% (3/4) of the wettable granules (WG) included in this evaluation had *in vitro* to *in vivo* ratios of less than 1, as did the only surfactant liquid (SL) and suspo-emulsion (SE) substances (one each).

Similar results were noted for the high dose group. Two formulations had extremely high absorption through *in vitro* skin, resulting in very large (greater than 29-fold) absorption ratios when compared to *in vivo* rat skin (Fig. 5B). Again, we identified no physicochemical properties or test method protocol components that could account for these outliers.

### 3.2 Comparison of dermal absorption in *in vitro* human and *in vitro* rat test methods

**Fig. 6:**
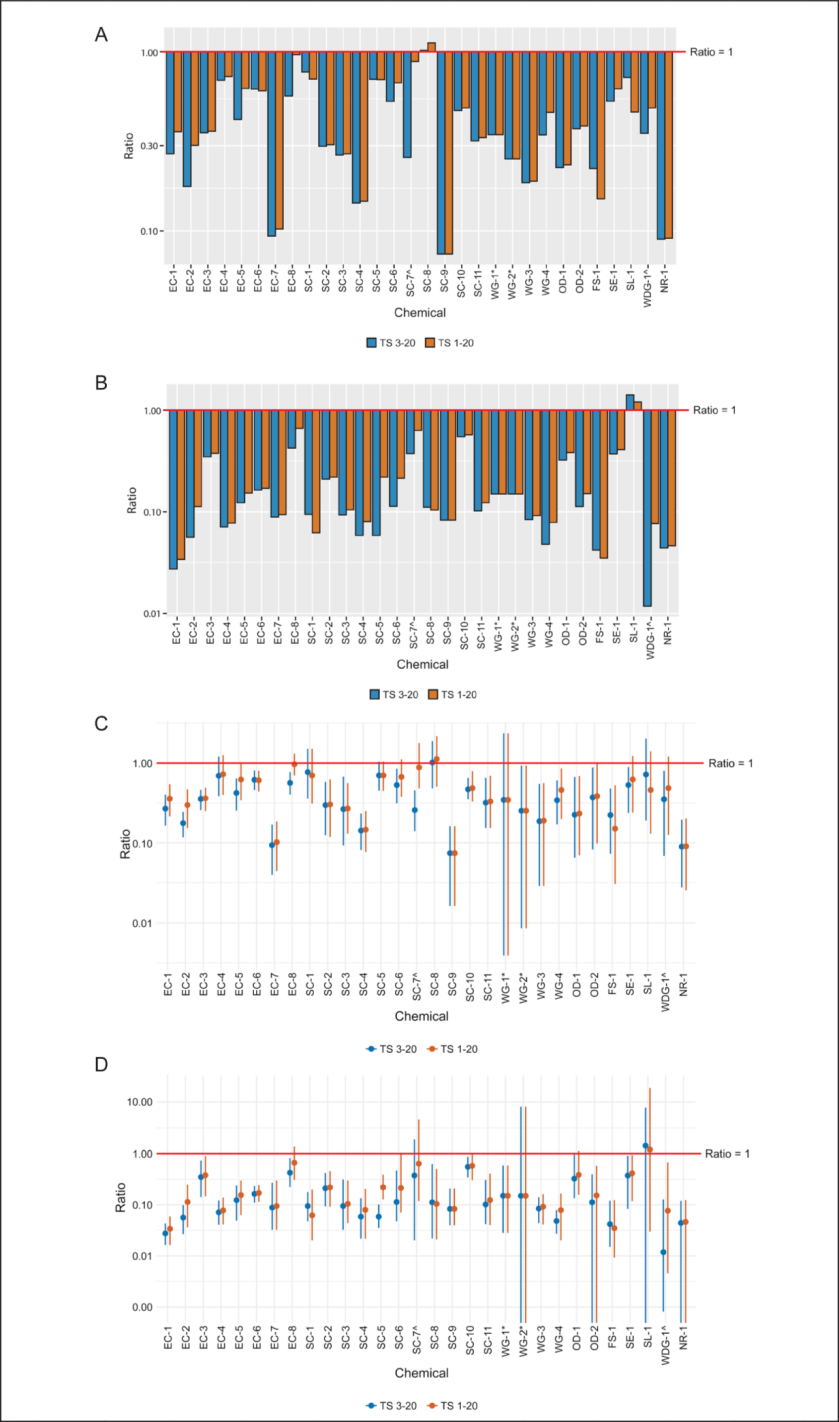
Dermal absorption time-matched comparisons at 24 h: *in vitro* human vs *in vitro* rat (A) Low dose group; (B) high dose group; (C) impact of variability on absorbance ratios, low dose group; (D) impact of variability on absorbance ratios, high dose group. ^ and * indicate formulations that include the same active ingredient in a different formulation.

In general, our data indicate that *in vitro* human absorption is approximately equal to or less than *in vitro* rat absorption. Time-matched comparisons in the current study demonstrated that the ratio of *in vitro* human potential absorption to *in vitro* rat potential absorption (calculated using tape strips 3–20) was approximately 1 or less for all 28 of the substances evaluated, with ratios ranging from 0.07 to 1.02 (Fig. 6A). Including all tape strips in the calculation of potential absorption had little impact. Using this more conservative approach, the absorption ratio was, again, approximately 1 or less for all 28 of the substances evaluated (Fig. 6A), with the highest ratio of 1.12 obtained for a suspension concentrate (SC) formulation. A similar trend was noted for the high dose comparisons (Fig. 6B). Similar to that seen for the *in vitro* human and *in vivo* rat test method comparisons, incorporating variability into the ratio calculations provided scenarios where the ratio of *in vitro* human to *in vitro* rat absorption could be much closer to 1 for all agrochemicals evaluated (Fig. 6C,D).

### 3.3 Comparison of dermal absorption in *in vitro* human and *in vivo* rat test methods

**Fig. 7:**
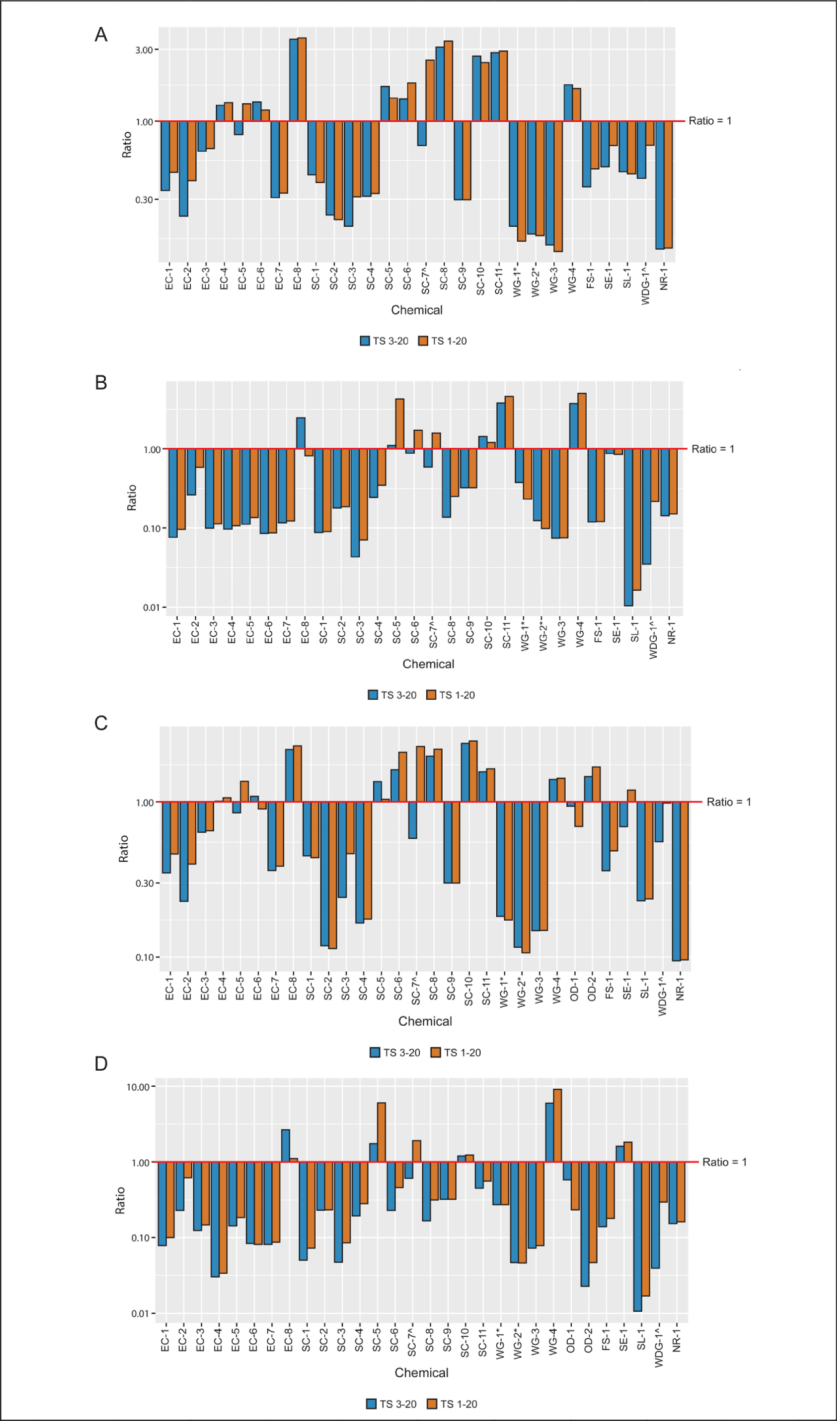
Dermal absorption comparisons: *in vitro* human add period to vs *in vivo* rat (A) Low dose groups time-matched at 24 h; (B) high dose groups time-matched at 24 h; (C) low dose groups longest time points (not time-matched); (D) high dose groups longest time points (not time-matched). ^ and * indicate formulations that include the same active ingredient in a different formulation.

As was observed in the comparison of the two *in vitro* approaches, absorption measured in the *in vitro* human assay was generally equal to or less than that measured in the *in vivo* rat assay. For 24 h time-matched comparisons of *in vitro* human and *in vivo* rat test method data, the ratio of human to rat potential absorption in the low dose groups (calculated using tape strips 3–20) was less than or equal to 1 for 68% (19/28) of the substances evaluated, with ratios ranging from 0.14 to 0.81 (Fig. 7A). For the remaining nine substances, ratios ranged from 1.27 to 3.50. As before, including all tape strips in the calculation of potential absorption had little impact, with 61% (17/28) of the substances evaluated having absorption ratios less than or equal to 1, with ratios ranging from 0.14 to 0.69 (Fig. 7A). For the remaining 11 substances, ratios ranged from 1.19 to 3.58. Similar trends were seen for the high dose comparisons (Fig. 7B).

Similar results were noted for ratios for *in vitro* human and *in vivo* rat potential absorption using the longest timepoint. In the low dose groups, ratios calculated using tape strips 3–20 were approximately 1 or less for 70% (21/30) of the substances, ranging from 0.09 to 1.01 (Fig. 7C). Of the substances with ratios greater than 1 in the longest time point analysis (ranging from 1.08 to 2.38), all but one also had ratios greater than 1 in the time-matched analysis. The one remaining substance did not have data at the matched timepoint for comparison. Including all tape strips in the calculation of potential absorption had little impact, with 63% (19/30) of the substances evaluated having absorption ratios of approximately 1 or less when using the more conservative approach (Fig. 7C). For the 11 remaining substances, ratios ranged from 1.3 to 2.5. Similar trends were seen for the high dose comparisons (Fig. 7D).

**Fig. 8:**
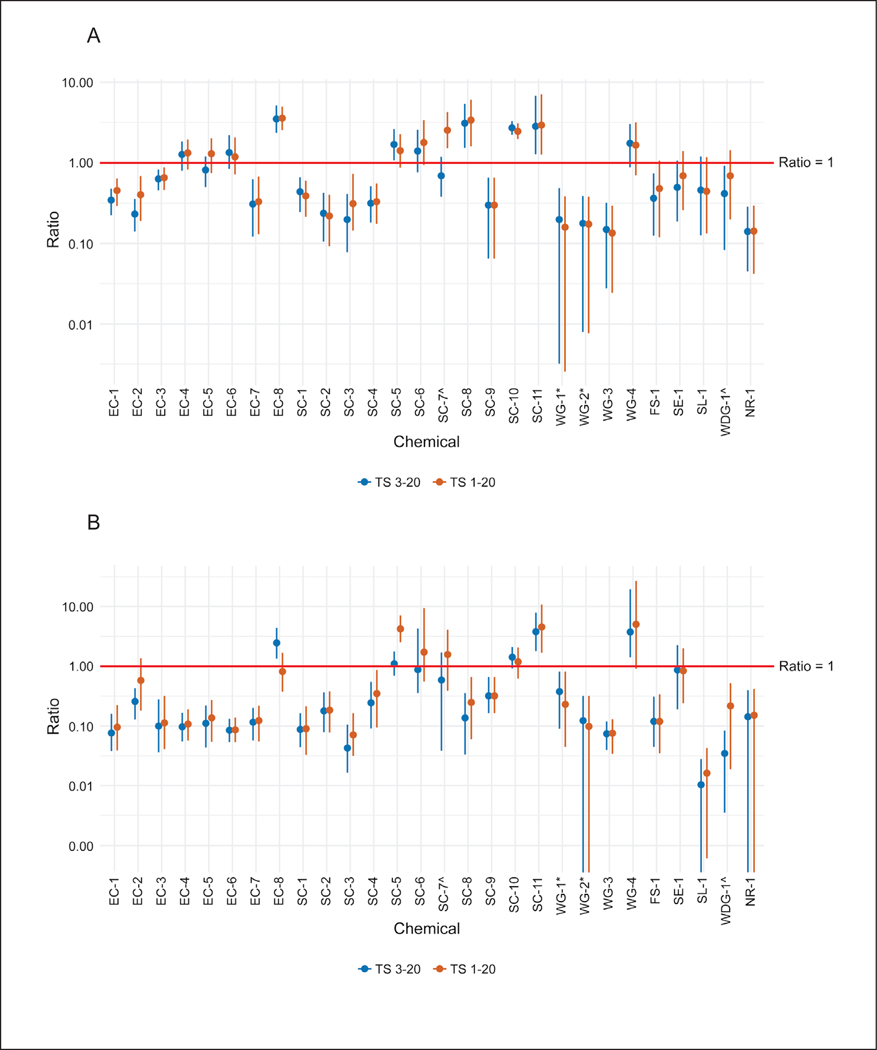
Impact of variability on absorbance ratios for *in vitro* human vs *in vivo* rat (A) Low dose group time-matched at 24 h; (B) high dose group time-matched at 24 h. ^ and * indicate formulations that include the same active ingredient in a different formulation.

Again, incorporating variability into the ratio calculations provided scenarios where the ratio of *in vitro* human to *in vivo* rat absorption could be much closer to 1 for all agrochemicals evaluated (Fig. 8).

### 3.4 Comparison of dermal absorption in *in vitro* human and the triple pack DAF

As noted above and shown in Figure 1, the ratio of human absorption to that of the triple pack DAF is mathematically equivalent to the ratio of the *in vitro* rat to *in vivo* rat, and thus the minimum and maximum human to triple pack ratios are visualized in Figure 3 and Figure 4. We have presented these results in Table 3 (24 h time-matched) and Table 4 (longest *in vivo* time point) to highlight the few instances where the DAF established solely based on *in vitro* human data provides a less conservative estimate of dermal absorption than the DAF derived from the triple pack (see shaded fields in Tab. 3 and 4).

When considering the low dose group in the 24 h time-matched dataset, 26% (8/30) of the substances had human *in vitro* DAF values that were less than the triple pack DAFs. However, all eight were within a 0.5-fold difference based on mean values. Furthermore, when we took variability into account and considered the upper end of possible responses, only one of those eight substances still had an absorbance value in the *in vitro* human study that was less than the triple pack DAF. For those substances with a human/TP DAF ratio of less than one, we also reviewed the absolute differences between DAFs calculated based on *in vitro* human data and the triple pack. Absolute differences at the low dose were found to be less than 3 for all except one substance (SC-1), which had relatively high dermal absorption measured by both methods (Tab. 3). Similarly, at the high dose, the differences in the values were less than 4 for all except one substance (SL-1) where penetration through *in vitro* human and rat skin was very low (Tab. 3).

We observed a similar trend in the dataset including the longest *in vivo* time points (Tab. 4). In these data, 23% (7/30) of substances had potential absorption values in the human *in vitro* assay that were less than the triple pack DAF. Again, for those substances with a human/TP DAF ratio of less than one, the absolute differences between DAFs calculated based on *in vitro* human data compared to the triple pack were very small (less than 4 and 3 for substances in the low and high dose groups, respectively) for all except three instances. This included the same two substances (one at both low and high doses (SL-1) and one at only the low dose (SC-1)) (Tab. 4).

## 4 Discussion

A NAFTA expert group concluded in 2008 that *in vitro* rat and/or *in vitro* human data alone were insufficient for predicting human dermal absorption of agrochemicals. The primary reason cited for this conclusion was the lack of detailed and standardized protocols for both methods and the resulting differences that might be seen across different laboratories. Since then, substantial efforts have been undertaken in the U.S. and internationally to provide guidance and standardized test guidelines that could be used to achieve more consistent results. The analysis we describe is based on data from triple pack studies conducted by industry stakeholders over the past decade according to procedures considered acceptable for regulatory use.

*In vitro* dermal absorption methods offer the possibility to reduce and refine (*in vitro* rat) or even completely replace (*in vitro* human) animal use for this endpoint. Additionally, moving away from relying on whole animal studies provides a more efficient and human-relevant approach to derive DAFs for human health risk assessment. The higher permeability of rat skin, which often provides an overestimate of dermal penetration potential, would be expected to represent a more conservative estimate of human dermal absorption. A central tenet of applying *in vitro* instead of *in vivo* data in a risk assessment is the assumption that absorption *in vitro* will be equivalent to or greater than that measured *in vivo*. The comparisons presented in this article were therefore performed to determine whether available data support potentially using *in vitro* absorbance data alone for DAF derivation for human health risk assessment of pesticides.

In most of the agrochemicals we evaluated, the ratio of *in vitro* to *in vivo* rat absorbance was greater than 1, indicating that the *in vitro* rat assay yields higher absorbance values and is therefore more conservative than the *in vivo* test method. Once the variability of each assay was applied to the calculated ratio, the likelihood of an absorbance ratio less than 1 was even further reduced, whereby only 1/30 substances still had a ratio of less than 1 for *in vitro* rat absorption: *in vivo* rat absorption. Our results also indicate that, in general, *in vitro* rat absorption is within 3-fold of *in vivo* rat absorption, suggesting that the *in vitro* estimate of dermal absorption is conservative without being overly sensitive as could be of concern to stakeholders. Somewhat surprisingly, there was little impact associated with calculating potential absorbance using all tape strips as compared to calculating potential absorbance excluding the first two tape strips due to the concern that they may include unabsorbable test substance. Although extensive details on the washing procedures used were not always available, this finding could have been due to a sufficiently rigorous washing procedure to limit the influence of the first two tape strips.

As expected, absorption by *in vitro* human skin was similar to or less than that observed in both *in vivo* and *in vitro* rat skin for all 30 agrochemical formulations evaluated. Most of the agrochemicals evaluated produced absorbance ratios of less than 1 when comparing *in vitro* human to *in vivo* rat skin, indicating higher dermal penetration for rat skin. Ratios that were greater than 1 were all less than 3. It should also be noted that, while absorption at the high dose is uniformly lower both in human and rat absorption, the ratio of low to high dose absorption *in vitro* in humans is much greater than that observed *in vitro* and *in vivo* in rats. This suggests that saturation of absorption kinetics was approached in human skin but not to the same extent in rat skin. However, it should also be noted that this difference is more pronounced *in vivo* than *in vitro*, which would explain the relative differences in the *in vitro* and *in vivo* rat when comparing high and low dose absorption (and consequently, human *in vitro* to triple pack DAF).

Many studies have shown that rat skin is more permeable than human skin (e.g., Bronaugh et al., 1982; van Ravenzwaay and Leibold, 2004), and our results are consistent with these. Given the increased density of hair follicles and decreased thickness of rodent skin relative to that of humans, one would assume that agrochemicals, like most other substances, would traverse rodent skin more readily than human skin, which provides a greater barrier. Again, there was little impact associated with inclusion of all tape strips vs excluding the first two when calculating absorption, regardless of comparing to *in vitro* or *in vivo* rat skin results. Just as in the rat test method comparisons, incorporating variability of each assay into the ratio calculations further increased the likelihood of an absorbance ratio approximating 1.

As described earlier, comparing the DAF obtained from the triple pack approach to a DAF obtained from the human *in vitro* study data alone is equivalent to a comparison of results obtained from the rat *in vitro* assay vs the rat *in vivo* assay. For most of the substances evaluated, the human *in vitro* assay provided a similar or higher estimate of dermal absorption than the triple pack. In those cases where the human *in vitro* assay provided a lower DAF value, the absolute difference of the values obtained from each approach (i.e., triple pack or human *in vitro* alone) were still lower than 4. The exceptions were limited to two substances noted at both the time-matched and longest time point comparisons.

These results are the culmination of efforts initiated in response to recommendations at the 2012 workshop (Sullivan et al., 2017) to compile and analyze the dataset envisioned by the NAFTA working group. Our retrospective evaluation of these three dermal absorption test methods indicates that *in vitro* rat data provide estimates of dermal absorptions that are at least as protective as *in vivo* rat data and thus could be considered adequate for use in establishing dermal absorption factors.

For human health risk assessment, *in vitro* tests using human skin would be preferable given their direct relevance to the species of interest (humans) and would also avoid the overestimation of dermal absorption using rat models given the higher permeability of rat skin as compared to human skin. And, of course, an *in vitro* human approach does not require animal-derived material and thus offers a complete replacement of animal use for this endpoint. However, *in vitro* rat studies still have utility when human *in vitro* data are not available. Our analyses have demonstrated that the *in vitro* rat assay provides a similar or more conservative estimate of human dermal absorption than the *in vivo* rat study. Due to the higher permeability of rat skin, the *in vitro* rat assay will likely overestimate the extent to which an agrochemical would be predicted to traverse human skin in a use case scenario (e.g., pesticide applicators in the field). Therefore, consistent with OECD and EFSA guidance, a DAF value from an *in vitro* rat study could be used as a preliminary estimate of dermal absorption for a pesticide when human *in vitro* data are not available to evaluate risk from dermal exposures.

## Figures and Tables

**Tab. 1:** Formulation types represented in this analysis

Formulation type	Number of formulations in the dataset
Emulsifiable concentrate (EC)	8
Flowable concentrate for seed treatment (FS)	1
Oil dispersion (OD)	2
Suspension concentrate (SC)	11
Soluble liquid (SL)	1
Water dispersible granules (WDG)	1
Suspo-emulsion (SE)	1
Wettable granules (WG)	1
Not reported (NR)	4
**Total**	**30**

**Tab. 2:** Data types extracted from study reports

*In vivo* rat	*In vitro* rat and human
● Formulation and study report ID ● Strain and sex ● Exposure duration ● Number of replicates ● Formulation type ● Concentration w/units ● Surface area treated and volume applied ● Occlusion ● Time point ● Amount of chemical potentially and directly absorbed ● Percent of chemical measured in urine, feces, carcass, blood, and tissues w/SD ● Tape strips 1 and 2 ● Tape strips 3–20 ● Cage wash ● Not absorbed ● Total recovery	● Formulation and study report ID ● Strain^a^ and sex ● Exposure duration ● Concentration w/units ● Skin preparation ● Skin site ● Skin thickness ● Tissue fresh/frozen ● Diffusion cell system ● Receptor fluid ● Number of replicates ● Number of donors^b^ ● Time point ● Formulation type ● Not absorbed ● Tape strips 1 and 2 ● Tape strips 3–20 ● Total recovery

a Only relevant to *in vitro* rat

b Only relevant to *in vitro* human

**Tab. 3:** Comparison of triple pack (TP) DAFs to estimates of dermal absorption from *in vitro* human assays: 24 h time-matched data

Index	TP DAF (low dose)	*In vitro* human (low dose)	Human/TP DAF (low dose)	Difference: *In vitro* human – TP DAF (low dose)	TP DAF (high dose)	*In vitro* human (high dose)	Human/TP DAF (high dose)	Difference: *In vitro* human – TP DAF (high dose)
EC-1	15.61	19.96	1.28	4.35	0.56	1.56	2.77	1
EC-2	4.96	6.5	1.31	1.54	0.37	1.697	4.65	1.327
EC-3	10.25	18.2	1.78	7.95	5.06	1.46	0.29	−3.6
EC-4	13.96	25.56	1.83	11.6	0.45	0.61	1.37	0.16
EC-5	9.73	18.85	1.94	9.12	1.1	1	0.91	−0.1
EC-6	12.28	26.5	2.16	14.22	1.53	0.8	0.52	−0.73
EC-7	1.84	6.04	3.29	4.2	0.79	1.04	1.32	0.25
EC-8	5.03	31.07	6.18	26.04	0.96	5.61	5.83	4.65
SC-1	47.1	26.788	0.57	**−20.31** **	0.28	0.259	0.93	−0.021
SC-2	2.98	2.38	0.8	−0.6	0.41	0.35	0.86	−0.06
SC-3	7.62	5.71	0.75	−1.91	0.28	0.13	0.46	−0.15
SC-4	0.78	1.72	2.21	0.94	0.07	0.28	4.17	0.21
SC-5	17.85	43.21	2.42	25.36	0.1	1.88	18.8	1.78
SC-6	3.62	9.57	2.64	5.95	0.05	0.36	7.78	0.31
SC-7^	5.36	14.34	2.67	8.98	0.58	0.92	1.58	0.34
SC-8	7.76	23.6	3.04	15.84	0.24	0.3	1.23	0.06
SC-9	1.08	4.32	4.02	3.24	0.04	0.17	3.89	0.13
SC-10	3.87	22.31	5.76	18.44	0.24	0.613	2.59	0.373
SC-11	0.59	5.29	8.94	4.7	0.03	1.1	37.31	1.07
WG-1*	5.37	3.1	0.58	−2.27	0.24	0.6	2.5	0.36
WG-2*	3.99	2.8	0.7	−1.19	1.22	1	0.82	−0.22
WG-3	1.32	1.06	0.8	−0.26	0.66	0.58	0.88	−0.08
WG-4	1.44	7.32	5.08	5.88	0.01	1	77.84	0.99
FS-1	0.89	1.457	1.63	0.567	0.06	0.172	2.85	0.112
SE-1	17	15.909	0.94	−1.091	1.69	3.985	2.36	2.295
SL-1	7.42	4.753	0.64	−2.667	27.61	0.204	0.01	**−27.41** **
WDG-1^	9.1	10.74	1.18	1.64	0.05	0.16	2.94	0.11
NR-1	0.91	1.42	1.57	0.51	0.15	0.49	3.23	0.34

Shaded text indicates *in vitro* human/triple pack DAF ratios < 1.

^ and * indicate formulations that include the same active ingredient in a different formulation

** absolute difference between *in vitro* human and triple pack DAF indicates triple pack DAF is much greater than *in vitro* human absorption.

**Tab. 4:** Comparison of triple pack (TP) DAFs to estimates of dermal absorption from *in vitro* human assays: longest *in vivo* time point

Index	TP DAF (low dose)	*In vitro* human (low dose)	Human/TP DAF (low dose)	Difference: *In vitro* human – TP DAF (low dose)	TP DAF (high dose)	*In vitro* human (high dose)	Human/TP DAF (high dose)	Difference: *In vitro* human – TP DAF (high dose)
EC-1	15.45	19.96	1.29	4.51	0.54	1.56	2.86	1.02
EC-2	5.03	6.5	1.29	1.47	0.42	1.697	4.08	1.277
EC-3	10.11	18.2	1.8	8.09	4.05	1.46	0.36	−2.59
EC-4	17.64	25.56	1.45	7.92	1.43	0.61	0.43	−0.82
EC-5	9.32	18.85	2.02	9.53	0.86	1	1.17	0.14
EC-6	15.18	26.5	1.75	11.32	1.56	0.8	0.51	−0.76
EC-7	1.57	6.04	3.84	4.47	1.12	1.04	0.93	−0.08
EC-8	8.1	31.07	3.83	22.97	0.89	5.61	6.27	4.72
SC-1	46.05	26.788	0.58	**−19.26** **	0.48	0.259	0.54	−0.221
SC-2	5.97	2.38	0.4	−3.59	0.32	0.35	1.1	0.03
SC-3	6.23	5.71	0.92	−0.52	0.26	0.13	0.5	−0.13
SC-4	1.48	1.72	1.16	0.24	0.08	0.28	3.31	0.2
SC-5	22.53	43.21	1.92	20.68	0.06	1.88	29.5	1.82
SC-6	3.15	9.57	3.03	6.42	0.18	0.36	2.03	0.18
SC-7^	6.33	14.34	2.26	8.01	0.56	0.92	1.64	0.36
SC-8	12.26	23.6	1.93	11.34	0.2	0.3	1.5	0.1
SC-9	1.08	4.32	4.02	3.24	0.04	0.17	3.89	0.13
SC-10	4.4	22.31	5.07	17.91	0.28	0.613	2.18	0.333
SC-11	1.08	5.29	4.91	4.21	0.25	1.1	4.43	0.85
WG-1*	5.82	3.1	0.53	−2.72	0.33	0.6	1.82	0.27
WG-2*	6.1	2.8	0.46	−3.3	3.23	1	0.31	−2.23
WG-3	1.33	1.06	0.8	−0.27	0.67	0.58	0.86	−0.09
WG-4	1.81	7.32	4.05	5.51	0.01	1	124.17	0.99
OD-1	0.68	2.84	4.16	2.16	0.58	1.04	1.8	0.46
OD-2	2.16	8.45	3.91	6.29	1.19	0.24	0.2	−0.95
FS-1	0.91	1.457	1.61	0.547	0.05	0.172	3.33	0.122
SE-1	12.26	15.909	1.3	3.649	0.92	3.985	4.34	3.065
SL-1	14.8	4.753	0.32	**−10.05** **	26.98	0.204	0.01	**−26.78** **
WDG-1^	6.83	10.74	1.57	3.91	0.05	0.16	3.33	0.11
NR-1	1.35	1.42	1.06	0.07	0.14	0.49	3.45	0.35

Shaded text indicates *in vitro* human/triple pack DAF ratios < 1.

^ and * indicate formulations that include the same active ingredient in a different formulation

** absolute difference between *in vitro* human and triple pack DAF indicates triple pack DAF is much greater than *in vitro* human absorption.

## Footnotes

1
https://ice.ntp.niehs.nih.gov/
